# Datasets for quantifying association between short-term exposure to maximum temperature and heatstroke-related ambulance dispatches in Japan: A time-stratified case-crossover design

**DOI:** 10.1016/j.dib.2025.111307

**Published:** 2025-01-14

**Authors:** Keita Wagatsuma

**Affiliations:** a Division of International Health (Public Health), Graduate School of Medical and Dental Sciences, Niigata University, Niigata, Japan; b Institute for Research Administration, Niigata University, Niigata, Japan

**Keywords:** Environmental health, Environmental epidemiology, Two-stage design, Case-cross over design, Heatstroke, Temperature, Distributed lag non-linear model, Meta-analysis

## Abstract

This study provides a comprehensive collection of open-access daily time-series datasets designed to quantify the association between short-term exposure to maximum temperature and heatstroke-related ambulance dispatches (HSAD) utilizing a two-stage time-stratified case-crossover design. The datasets include daily records of HSAD, maximum temperature (°C), and relative humidity (%) for the summer months (i.e., June to September) from 2015 to 2019, spanning all 47 Japanese prefectures and covering the entire country. These datasets are well-suited for time-series regression analyses to estimate location-specific maximum temperature-HSAD associations in Japan. The availability of data from multiple locations also facilitates the of regional differences by aggregating location-specific associations within broader geographical areas. This resource is intended to support researchers, educators, and students in leveraging time-series analysis for research and educational purposes.

Specifications Table

**Table:** 

Subject	Global and Planetary Change; Health, Toxicology and Mutagenesis; Epidemiology.
Specific subject area	Quantifying the association between short-term exposure to maximum temperature and heatstroke-related ambulance dispatches with a two-stage time-stratified case-crossover design.
Type of data	Raw, Analysed, Filtered
Data collection	Daily data were collected during the summer season (i.e., June to September) from 2015 to 2019 for all 47 Japanese prefectures. These datasets included daily counts of heatstroke-related ambulance dispatches, as well as records of prefecture-specific maximum temperature (°C) and relative humidity (%).
Data source location	All 47 Japanese prefectures (i.e., Hokkaido, Aomori, Iwate, Miyagi, Akita, Yamagata, Fukushima, Ibaraki, Tochigi, Gunma, Saitama, Chiba, Tokyo, Kanagawa, Niigata, Toyama, Ishikawa, Fukui, Yamanashi, Nagano, Gifu, Shizuoka, Aichi, Mie, Shiga, Kyoto, Osaka, Hyogo, Nara, Wakayama, Tottori, Shimane, Okayama, Hiroshima, Yamaguchi, Tokushima, Kagawa, Ehime, Kochi, Fukuoka, Saga, Nagasaki, Kumamoto, Oita, Miyazaki, Kagoshima, and Okinawa).
Data accessibility	Repository name: Zenodo Data identification number: 10.5281/zenodo.14560225 Direct URL to data: https://zenodo.org/records/14560225

## 1 Value of the Data

• This time-series dataset facilitates a comprehensive assessment of exposure-response relationships across multiple locations utilizing a two-stage time-stratified case-crossover design
• The dataset also has educational value, illustrating their application to studies involving maximum temperatures and heatstroke-related ambulance dispatches through time-series regression models, distributed lag non-linear models, and meta-analysis
• These examples offer foundational knowledge of time-series analysis in environmental epidemiology and enhance the reproducibility of research findings.

## 2 Background

Environmental epidemiology research frequently evaluates the short-term associations between ambient environmental stressors (e.g., temperature) and health outcomes (e.g., mortality) utilizing time-series data from multiple geographical locations [1]. In recent years, the two-stage time-stratified case-crossover design has become the prevailing analytical framework for these statistical assessments [2]. This approach comprises two sequential steps: first, location-specific exposure-response relationships are estimated while controlling for relevant potential confounders; second, the location-specific estimates are synthesized using meta-analytic techniques, integrating meta-predictors to capture location-specific characteristics [3]. The principal advantage of this case-crossover design lies in its ability to control for observed and unobserved time-invariant individual confounders, such as age, education, health care access, socio-economic status, and other related factors, by design [2].

This data article offers a contemporary introduction to the two-stage time-stratified case-crossover design for investigating environmental stressors and provides a practical guide to its application in environmental epidemiology. It features a detailed compilation of openly accessible time-series datasets tailored to quantify the association between short-term exposure to maximum temperature and heatstroke-related ambulance dispatches (HSAD) in Japan, as illustrated through a case study [[4], [5], [6]]. This resource is designed to support researchers, educators, and students in employing time-series analysis for research purposes and educational endeavours.

## 3 Data Description

### 3.1 Study setting

Situated between latitudes 26°N and 43°N and longitudes 127°E and 141°E, Japan is in the Western Pacific Region. The country consists of all 47 prefectures (i.e., Hokkaido, Aomori, Iwate, Miyagi, Akita, Yamagata, Fukushima, Ibaraki, Tochigi, Gunma, Saitama, Chiba, Tokyo, Kanagawa, Niigata, Toyama, Ishikawa, Fukui, Yamanashi, Nagano, Gifu, Shizuoka, Aichi, Mie, Shiga, Kyoto, Osaka, Hyogo, Nara, Wakayama, Tottori, Shimane, Okayama, Hiroshima, Yamaguchi, Tokushima, Kagawa, Ehime, Kochi, Fukuoka, Saga, Nagasaki, Kumamoto, Oita, Miyazaki, Kagoshima, and Okinawa) and predominantly experiences a temperate climate with four distinct seasons, including hot, humid summers (June to August) and cold, dry winters (December to February) .

### 3.2 Epidemiological data

We collected daily HSAD counts data in the summer season (June to September) from 2015 to 2019 of all 47 Japanese prefectures from the Fire and Disaster Management Agency of the Ministry of Internal Affairs (FDMA) database (https://www.fdma.go.jp/). Heat stroke-related diagnoses were classified based on the International Classification of Diseases (ICD) 10 codes. More specifically, the following diagnostic categories were included and consolidated under heat stroke: “heatstroke and sun stroke” (T67.0), “heat syncope” (T67.1), “heat cramp” (T67.2), “heat exhaustion, anhidrotic” (T67.3), “heat fatigue, unspecified” (T67.5), “heat fatigue, transient” (T67.6), “heat edema” (T67.7), and “other effects of heat and light” (T67.8) [[4], [5], [6]]. Meteorological data, including daily maximum temperature (°C) and relative humidity (%), were obtained for the same period from a single monitoring station managed by the Japan Meteorological Agency (JMA) (https://www.jma.go.jp/jma/index.html). The dataset included calendar variables such as date, year, month, day of the week, and national public holidays. All data were anonymized, aggregated, sourced from publicly available databases, and exempt from ethical approval requirements.

## 4 Experimental Design, Materials and Methods

The time-series regression design is a widely used analytical method in environmental epidemiology for examining short-term associations between environmental exposures and health outcomes [1]. In this study, we quantified the association between short-term exposure to maximum temperature and HSAD in Japan. Specifically, we conducted a two-stage time-series analysis to model multi-location data, incorporating time-series datasets for all 47 Japanese prefectures [3]. In the first stage, we estimated the exposure-response relationship in each prefecture. We then pooled the prefecture-specific effect estimates to obtain an overall mean in the second stage using a meta-analysis. Between June and September from 2015 to 2020, a total of 300,528 HSAD cases were documented across 47 Japanese prefectures (Table 1). During this period, the mean daily HSAD cases began increasing in June (mean: 2.7 cases [standard deviation: 4.4 cases]), reached a peak in July (18.8 cases [31.4 cases]) and August (18.3 cases [28.2 cases]), and then declined in September (2.8 cases [7.3 cases]). The maximum recorded temperature during the study period occurred in August at 32.1°C, while the highest relative humidity was observed in September at 76.5%.

**Table 1:** Annual summary statistics of daily counts of HSAD and meteorological variables in four Japanese prefectures during summer period (from June to September) in 2015–2019.

Variables	June	July	August	September	Overall
HSAD (cases per day)	2.7 (4.4)	18.8 (31.4)	18.3 (28.2)	2.8 (7.3)	10.6 (22.9)
Maximum temperature (°C)	26.4 (3.3)	30.6 (3.7)	32.1 (3.4)	27.6 (3.4)	29.1 (4.1)
Relative humidity (%)	74.2 (12.0)	77.4 (9.2)	74.3 (9.3)	76.5 (10.4)	75.5 (10.3)

Notes: The cell values represent the mean, with the standard deviation shown in parentheses. Abbreviations: HSAD, heatstroke-related ambulance dispatches.

In the first stage, we adopted a time-stratified case-crossover study design to analyze prefectural data using a conditional quasi-Poisson regression model combined with a distributed lag non-linear model (DLNM) [2,7]. Adjustments for seasonality, long-term trends, and day of the week effects were achieved through time stratification defined by a three-way interaction term of year, month, and day of the week. This case-crossover design is useful for controlling for individual characteristics which are unlikely to change within the small-time window, such as demographic characteristics, e.g., sex and education and living habits, e.g., smoking and drinking [2]. To investigate the non-linear and multi-delayed relationship between maximum temperature and HSAD, we applied a cross-basis function within the DLNM framework. This cross-basis function utilized a natural cubic B-spline for the exposure-response association, with internal knots placed at the 25^th^, 50^th^, and 75^th^ percentiles of maximum temperature, and another natural cubic B-spline for the lag-response association, spanning a 7-day lag period with three equally spaced knots on a logarithmic scale [8]. Other potential confounding factors were also addressed by incorporating relative humidity, modeled as a natural cubic B-spline with three degrees of freedom, and by accounting for national public holidays.

In the second stage, a multivariate meta-analysis based on a random-effects model was used to pool the prefecture-specific estimates from the first-stage analysis [9]. We included the prefecture-specific latitude in the model as meta-predictor to investigate heterogenicity. To quantify heterogeneity, we calculated the *I*^2^ statistics and performed the Cochran's *Q* test. We estimated the relative risks (RRs) and corresponding 95% confidence intervals (CIs) using the median maximum temperature, i.e., 29.3°C, as the reference value. The two-tailed *p-*values less than 0.05 were considered statistically significant. All statistical analyses were performed using R version 4.1.0 (R Foundation for Statistical Computing, Vienna, Austria) with the packages “*gnm*”, “*dlnm*”, and “*mixmeta*”. The R code for analysing the example is available in the repository of Zenodo.

**Fig. 1:**
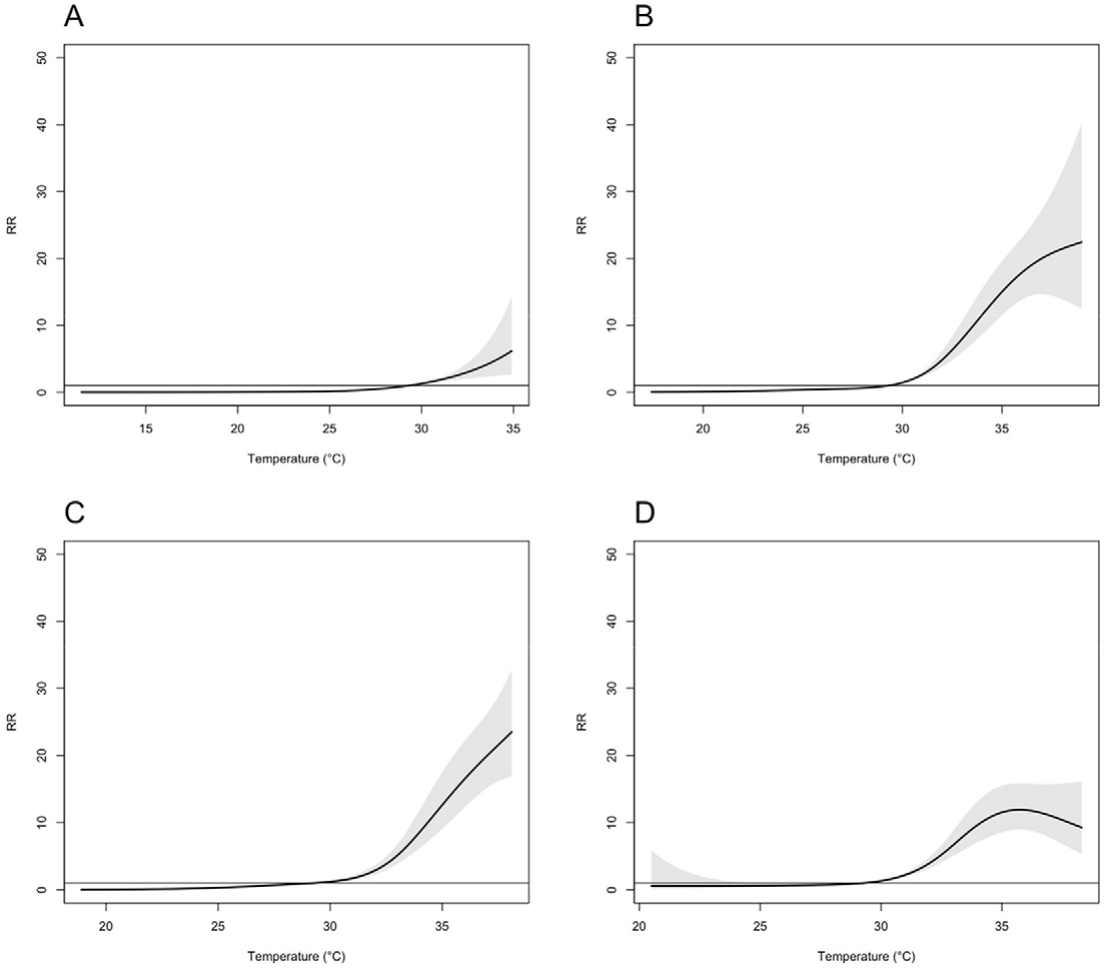
Pooled estimates of overall lag-cumulative associations between maximum temperature and HSAD with 95% confidence intervals (shaded grey) for A: Hokkaido, B: Tokyo, C: Osaka, and D: Fukuoka. Centred at the median value of maximum temperature at 29.3°C. Abbreviations: RR, relative risk; HSAD, heatstroke-related ambulance dispatches.

Fig. 1 illustrates the overall cumulative exposure-response curves between maximum temperature and HSAD across for four major Japanese prefectures. Overall, a J-shaped association was evident in all prefectures, including Hokkaido, Tokyo, Osaka, and Fukuoka, with elevated RR values for heat temperatures. Notably, Tokyo and Osaka demonstrated exceptionally high RR values. Conversely, in Fukuoka, the RR for heat temperatures showed a tendency to decrease at maximum temperatures exceedingly approximately 35°C. The corresponding graphs for all 47 Japanese prefectures are reported in the supplementary material (Fig. S1).

**Fig. 2:**
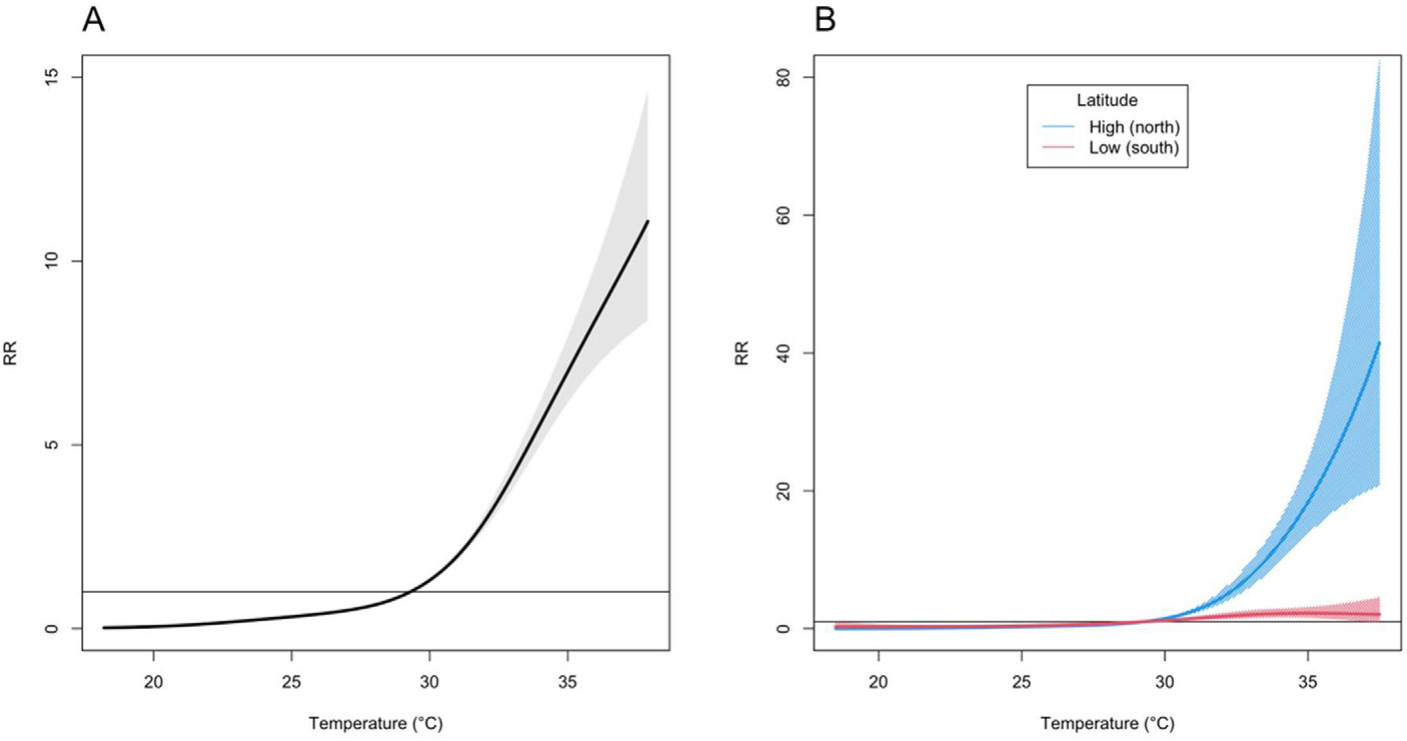
Pooled estimates of overall lag-cumulative associations between maximum temperature and HSAD with 95% confidence intervals in all 47 Japanese prefectures. Notes: The panel A displays the average heat-HSAD curve derived from the multivariate meta-analysis, incorporating only intercepts and no predictors. The panel B illustrates the effect modification by latitude, as predicted from the full multivariate meta-regression, based on the 10^th^ to 90^th^ percentile values of the prefecture-specific meta-variable. Abbreviations: RR, relative risk; HSAD, heatstroke-related ambulance dispatches.

Fig. 2 depicts the pooled estimates of overall cumulative exposure-response relationship between maximum temperature and HSAD over a 7-day lag period for all 47 Japanese prefectures. A J-shaped association was also evident nationwide. At the 95^th^ percentile of the maximum temperature distribution (i.e., 37.0°C), the heat effect yielded an RR of 9.7 (95% CI: 7.8–12.1) compared to 29.3°C (left panel in Fig. 2). A meta-analysis revealed substantial heterogeneity in the maximum temperature-HSAD associations across prefectures, with an *I*² of 82.2% and a highly significant Cochran's *Q* test (*p*-value < 0.001). To investigate this variability, latitude was included as a predictor in multivariate meta-regressions (Table 2). In a univariable model, latitude emerged as a significant modifier of the maximum temperature-HSAD associations, with northern prefectures showing significantly higher risks at elevated maximum temperatures (right panel in Fig. 2). The likelihood ratio test confirmed that latitude accounted for the observed heterogeneity in HSAD across prefectures (*p*-value < 0.001).

**Table 2:** Random-effects meta-regression models in the second-stage analysis.

Meta-predictor	AIC	BIC	*I*^2^ statistics (%)	Cochran's *Q* test (*p*-value)	LR test (*p*-value)
Intercept only	560.6	606.0	79.6	< 0.0001	
Latitude	525.3	583.6	67.3	< 0.0001	< 0.0001

Abbreviations: AIC, Akaike information criterion; BIC, Bayesian information criterion; LR, likelihood ratio.

This case study demonstrated the applicability of the time-series datasets to estimate the exposure-response association between environmental stressors and health outcomes using a two-stage time-stratified case-crossover design. Furthermore, the availability of time-series data from multiple geographical locations also enabled the exploration of geographical differences by pooling location-specific associations within geographical regions. Recent advancements in statistical techniques for time-series analysis in environmental epidemiology have led to the creation of various evaluation frameworks. For instance, the time-stratified design in this study can be expanded into a space-time-stratified case-crossover design, allowing multilevel data to be analysed in a single step as an alternative to the conventional two-stage design [2,10]. In contrast, when individual exposure measurements are available, the recently introduced case-time-series design offers a flexible and computationally efficient approach for addressing temporal dependencies while avoiding potential biases from between-area comparisons [11]. Traditionally, spatial and spatio-temporal modelling analyses have utilized Bayesian hierarchical models, such as the integrated nested Laplace approximation [12]. Although these methods provide a rigorous framework for accounting for spatial correlations and geographically varying risks, their high computational requirements often limit their applicability to large datasets or complex relationships. Selecting the appropriate statistical methodology based on the characteristics of the time-series data set and evaluating its applicability is essential for researchers. Nonetheless, simulation studies remain necessary to investigate the performance and suitability of various statistical models.

## Limitations

To our knowledge, this study is the first to model the maximum temperature-HSAD associations between in Japan. However, several limitations should be acknowledged. First, the time-series analysis was conducted within the framework of an ecological study, which inherently limits causal inference. Second, the study's scope was confined to 47 Japanese prefectures, which restricts the generalizability of the findings to other regions or countries. Third, the lack of individual-level data prevented stratified analyses by age, sex, illness severity, or incident place, thereby precluding a comprehensive assessment of vulnerability among subgroups.

## Ethics Statement

The author has read and follow the ethical requirements for publica on in Data in Brief and confirming that the current work does not involve human subjects, animal experiments, or any data collected from social media platforms.

## CRediT Author Statement

**Keita Wagatsuma:** Conceptualization, Writing – original draft, Software, Methodology.

## Data Availability

ZenodoDatasets for quantifying association between short-term exposure to maximum temperature and heatstroke-related ambulance dispatches in Japan: a time-stratified case-crossover design (Original data).

## Appendix Supplementary materials

Image, application 1
