# Supplementary material for: Datasets for quantifying association between short-term exposure to maximum temperature and heatstroke-related ambulance dispatches in Japan: A time-stratified case-crossover design
Source: Data Brief. 2025 Jan 14;59:111307. doi: 10.1016/j.dib.2025.111307 (PMC11804774; doi:10.1016/j.dib.2025.111307)
Supplement: Supplementary file 1 [file mmc1.docx]

# Supplementary material


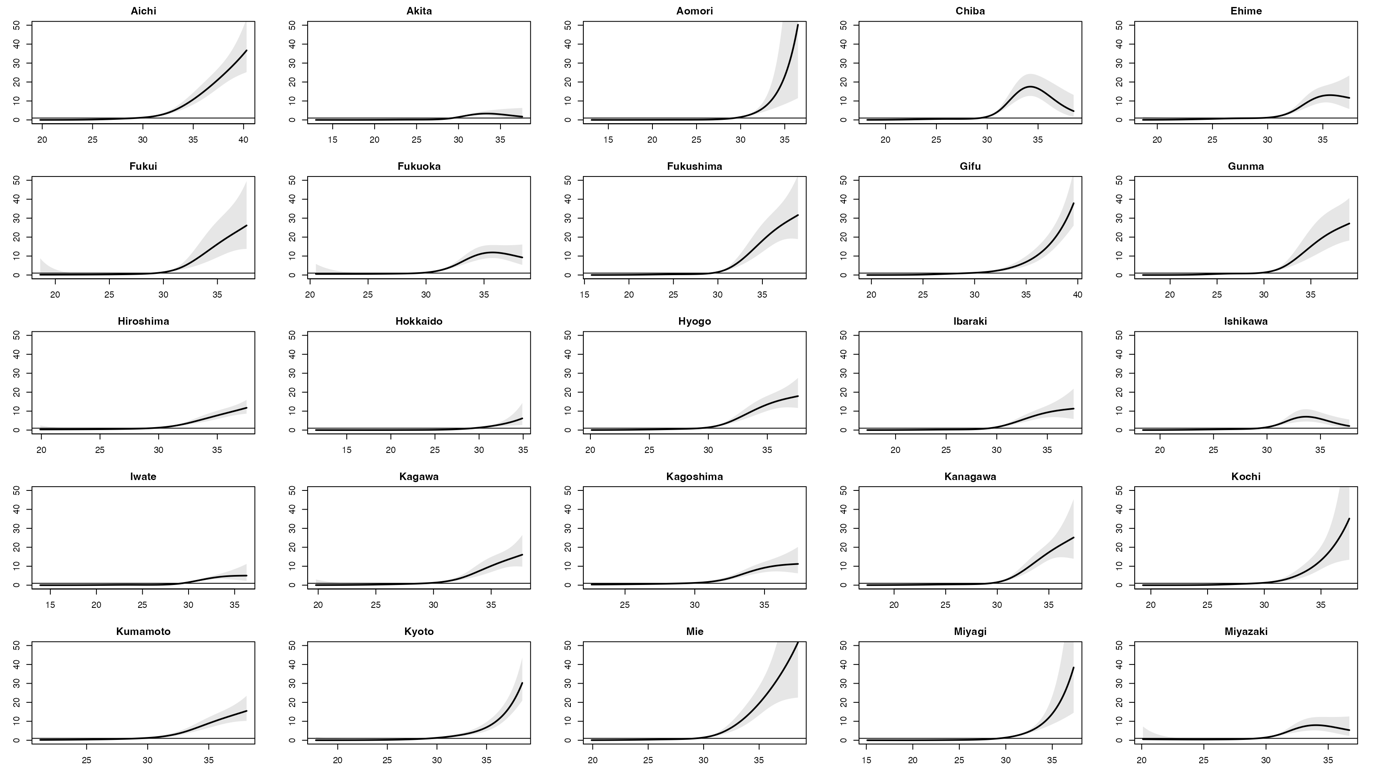

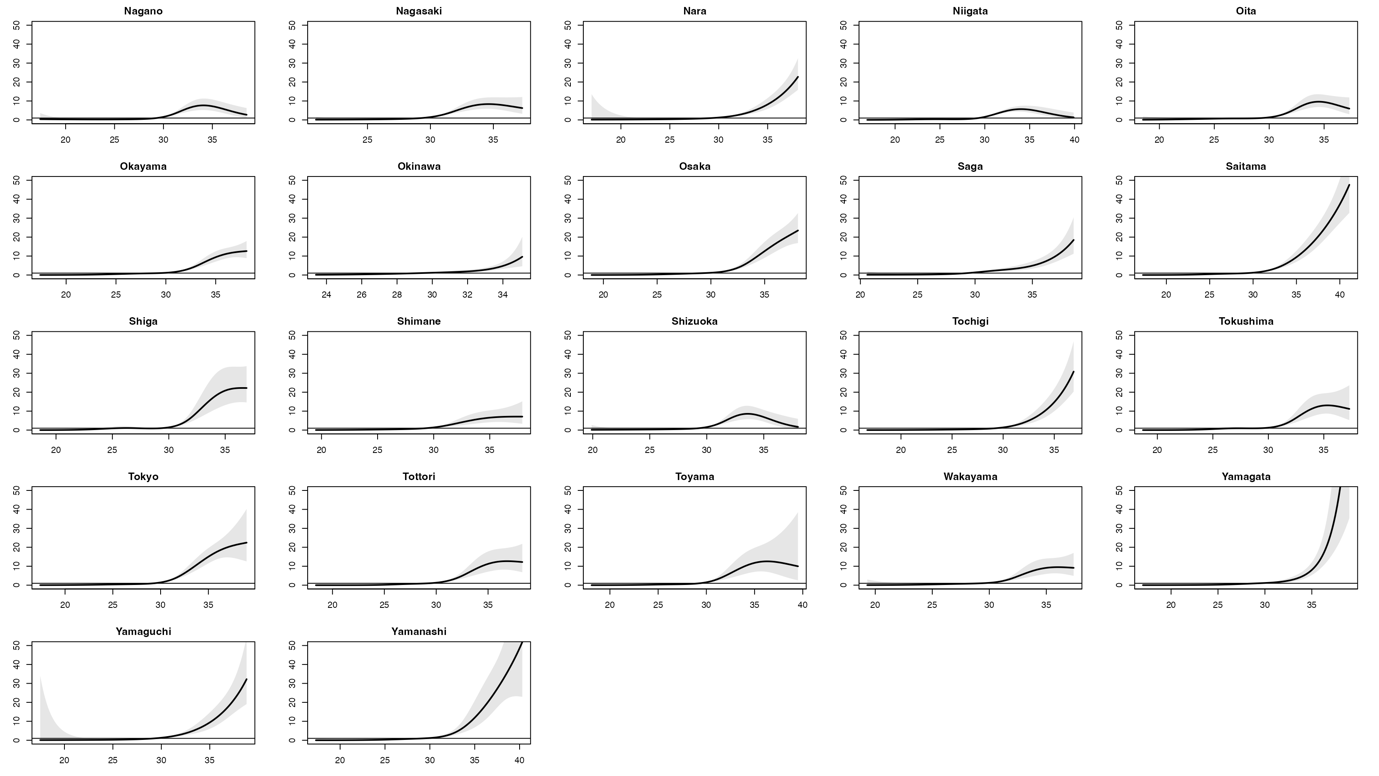


Fig. S1. Pooled estimates of overall lag-cumulative associations between maximum temperature and HSAD with 95% confidence intervals (shaded grey) for 47 Japanese prefectures. Centred at the median value of maximum temperature at 29.3 °C. Abbreviations: RR, relative risk; HSAD, heatstroke-related ambulance dispatches.
